# Streamlining Endoscopy Cleaning: The Impact of a New Detergent on Time and Water Use

**DOI:** 10.3390/jmahp13020023

**Published:** 2025-05-16

**Authors:** Joshua Hicks, Mutsa Mutowo

**Affiliations:** 1 Olympus KeyMed Group Ltd., Southend-on-Sea SS2 5QH, UK; 2 Olympus Australia, Notting Hill, Melbourne, VIC 3168, Australia; 3 Macquarie University Centre for the Health Economy, Sydney, NSW 2113, Australia

**Keywords:** flexible endoscopes, water consumption, manual cleaning, operational efficiency, decontamination, sustainability, carbon emissions, healthcare, resource conservation

## Abstract

Reprocessing reusable flexible endoscopes is resource-intensive and involves high water consumption. This study evaluated the impact of replacing a standard detergent with EndoPreZyme™, a novel detergent, at Blackpool Teaching Hospitals NHS Foundation Trust. We assessed manual cleaning times, water usage, costs, and technician experiences. A direct observational time system analysis was conducted over two one-week periods to record technician tasks before and after implementing EndoPreZyme™, allowing for the omission of the final rinse after manual cleaning. Technician surveys captured user experiences during the transition. The results showed that removing the final rinse after manual cleaning reduced water consumption by 25 litres per endoscope, resulting in an estimated saving of 725,000 L annually. The average manual cleaning time decreased from 13 min 10.2 s to 11 min 10.7 s—a reduction of 1 min 59.5 s per endoscope (15%). This efficiency gain translated to approximately 962.9 fewer technician hours being required annually for manual cleaning. Cost analysis revealed a slight per-endoscope cost reduction (GBP 4.88 vs. GBP 4.90). Technicians reported improved productivity, reduced workload, and an awareness of water conservation. These findings demonstrate that EndoPreZyme™ supports NHS sustainability goals by decreasing water usage and enhancing operational efficiency in healthcare delivery.

## 1. Introduction

The National Health Service (NHS) is responsible for approximately 40% of public sector carbon emissions, with healthcare accounting for 4–5% of this total [1]. As one of the first national healthcare systems in the world to commit to achieving net-zero emissions by 2040 [2], the NHS faces significant pressure to reduce its environmental impact.

Each year, the NHS consumes 50 billion litres of water [3], resulting in considerable expenses associated with high water costs for its hospitals, clinics, and other medical facilities [4]. While estimates of water expenditure vary by region, hospital size, and facility usage, they are estimated to exceed GBP 80 million [5]. In response to economic and environmental challenges, best practices in efficiency and innovation are being implemented to minimise water usage and waste [6].

The 2021 strategy paper titled “Delivering a Net Zero NHS” highlighted the importance of water management within healthcare. NHS England has pledged to reduce water usage by 40% by 2025 and will continuously monitor water consumption to identify increases and opportunities for improvement [7].

### 1.1. Water Usage and Environmental Impact of Endoscopy Reprocessing

Since the 1850s, endoscopy has involved the insertion of a visualisation device directly into an organ or internal cavity [8]. These procedures are performed in various healthcare settings to examine different parts of the body, including the lungs (bronchoscopy), colon (colonoscopy), bladder and urethra (cystoscopy and ureteroscopy), upper gastrointestinal tract (esophagogastroduodenoscopy), and upper airway (Rhinolaryngoscopy). Most flexible endoscopes are reusable and require thorough cleaning and reprocessing [9].

The resource-intensive process of reprocessing these reusable flexible endoscopes has notable environmental consequences, particularly regarding water usage. To prevent infections, reusable flexible endoscopes undergo meticulous cleaning and disinfection. This multi-step process requires significant amounts of tap, filtered, or deionised water, as well as detergents, disinfectants, heat, and electricity [10,11].

### 1.2. Challenges in Meeting Reprocessing Demand

The demand for endoscopic services in the UK has doubled in the past five years, largely due to an ageing population, an increase in gastrointestinal diseases, and the implementation of the NHS Bowel Cancer Screening Programme in England [12,13]. As a result, healthcare institutions are under significant pressure to efficiently and effectively reprocess reusable flexible endoscopes to accommodate the growing volume of treatments.

Technicians in decontamination units are responsible for overseeing infection control, ensuring quality assurance, and maintaining patient safety during the reprocessing of reusable flexible endoscopes. They must manage comprehensive cleaning while adhering to short turnaround times in high-volume clinical settings [14].

### 1.3. The Significance of Manual Cleaning

The seven steps involved in the reprocessing of reusable flexible endoscopes are as follows: pre-cleaning, manual cleaning, rinsing, disinfection, a second rinsing, drying, and storage [15]. These steps are consistent across various guidelines [16,17,18,19,20]. The reprocessing of reusable flexible endoscopes begins with manual cleaning, which is crucial because inadequate cleaning can compromise high-level disinfection [21,22,23]. The cost of manually cleaning a single reusable flexible endoscope has been reported to range from GBP 8.60 to GBP 28.73, which includes materials and personnel time [24,25].

Before manual cleaning, a leak test must be conducted, and all detachable components should be disassembled. The external surfaces should be cleaned using an appropriate neutral detergent along with line-free cloths, sponges, or single-use brushes. Accessible channels should be flushed with a suitable flushing device and brushed to remove debris and other contaminants [15].

Following manual cleaning, the reusable flexible endoscopes should be rinsed with clean water to ensure the removal of detergent residues [24]. This step, often referred to as the “final rinse step after manual cleaning”, is performed before the reusable flexible endoscope is processed in an Endoscope Washer Disinfector (EWD). The final rinse after manual cleaning is a standard part of the manual cleaning process and is essential for maintaining the effectiveness of disinfection, thus maintaining patient safety.

EndoPreZyme^TM^ is a neutral, enzymatic detergent manufactured by Chemische Fabrik Dr. Weigert GmbH & Co. KG (Hamburg, Germany) and distributed by Olympus Winter & Ibe GmbH (Hamburg, Germany). It is specifically designed for the manual cleaning of reusable flexible endoscopes prior to automated cleaning in an EWD.

The use of EndoPreZyme^TM^ allows for the omission of the final rinse after manual cleaning, but this is only applicable when the reusable flexible endoscope is subsequently reprocessed in a validated EWD [26,27].

The manufacturer, Chemische Fabrik Dr. Weigert GmbH & Co. KG, identifies several validated EWDs, which include the ETD3 PAA/ETD3 GA, ETD4 PAA/ETD4 GA, miniETD2 PAA/miniETD2 GA, ETD Double, and ETD Mini.

It is important to note that while EndoPreZyme™ can be used during manual cleaning when a validated EWD is not available, the final rinse after manual cleaning is mandatory in such cases, followed by subsequent reprocessing in an EWD [26,27]. This requirement applies whether EndoPreZyme™ is used as a standalone detergent or when reprocessing will not continue in a validated EWD.

Furthermore, the option to omit the final rinse after manual cleaning is supported by the manufacturer’s Instructions for Use (IFU) and is based primarily on the low-foaming characteristics of EndoPreZyme™, rather than its enzymatic properties. Excessive foaming during automated reprocessing can disrupt cleaning cycles; however, compatibility tests conducted under worst-case scenarios—such as a threefold detergent overdose and inlet water temperatures as low as 5 °C—have shown that residual EndoPreZyme™ does not negatively impact the cleaning or disinfection performance of validated EWDs [26,27].

Eliminating the final rinse after manual cleaning with EndoPreZyme™ may reduce water usage, shorten the manual cleaning time, and help healthcare facilities meet the demand for endoscopic procedures. However, it is important to note that no studies have quantified these benefits.

### 1.4. EndoPreZyme™ Will Henceforth Be Referred to as the ‘New Detergent’

This observational study investigates the impact of replacing the standard detergent with a new detergent during manual cleaning, which is a crucial step in the process of cleaning reusable flexible endoscopes. This study investigates the impact of eliminating the final rinse after manual cleaning on water usage, manual cleaning time, efficiency, resource costs, and the experiences of decontamination technicians at the Endoscopy Decontamination Unit (EDU) at Blackpool Teaching Hospitals NHS Foundation Trust. This study aims to enhance the operational efficiency of manual cleaning while meeting the sustainability objectives of the NHS.

## 2. Materials and Methods

### 2.1. Design

This was a single-centre, direct observational time system (DOTS) analysis conducted over two separate one-week periods in the EDU of Blackpool Teaching Hospitals NHS Foundation Trust, Lancashire, England.

### 2.2. Setting

Blackpool Teaching Hospitals NHS Foundation Trust was selected as the study centre due to its transition from its standard detergent to the new detergent during the study period. This transition enabled a direct comparison of the manual cleaning process before and after the change in detergent.

The trust operates a centralised EDU equipped with three double-bowl sinks, one single-bowl sink, and seven ETD Double units (manufactured by Olympus Winter & Ibe GmbH). The ETD Double is a pass-through EWD that allows for the simultaneous reprocessing of up to three reusable flexible endoscopes.

### 2.3. Observations

Observations of 13 Band 2 decontamination technicians who were responsible for manually cleaning reusable flexible endoscopes were conducted during the study.

Two independent observers, both certified industrial engineers specialising in work quantification and Performance Assessment and Comparison Evaluation (PACE) ratings, conducted these observations. They adhered to the standards established by the Institute of Management Services and participated in an annual PACE rating clinic to maintain their certification. Before their observations, each observer was independently given an overview of the manual cleaning process at the study facility.

Each observer was assigned to a specific study phase: one documented the manual cleaning process using the standard detergent, while the other observed the use of the new detergent. To ensure consistency in data collection and task classification across the study phases, a handover discussion took place before transitioning from the standard to the new detergent. During this discussion, the observers reviewed task categorisation, workflow definitions, and data recording categories to ensure that both datasets were aligned.

**Figure 1:**
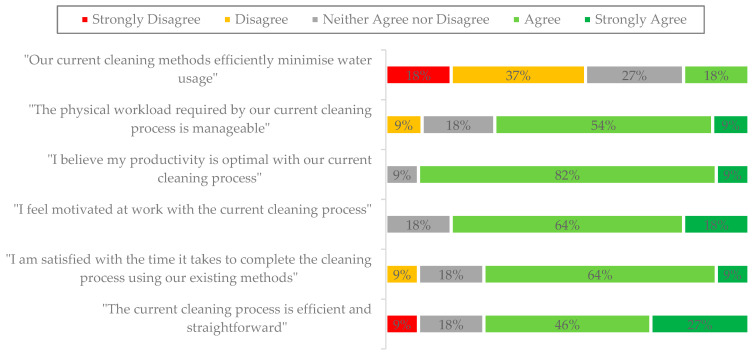
Impact of the standard detergent on the decontamination technicians’ experiences.

The observers followed a structured observation protocol to systematically record each step of the manual cleaning process. This process began when the reusable flexible endoscope entered the ‘Dirty Room’ for leak testing, manual cleaning, and visual inspection, and concluded when the reusable flexible endoscope was placed in the EWD (see Figure 1).

Although formal inter-rater reliability measures were not calculated, the use of standardised observation protocols and predefined data categories ensured consistency in data collection, minimising variability in the comparison between the two detergents.

Observations were conducted on weekdays between 9 am and 5 pm over two intervals: 12–15 March and 18 March 2024, and 13–19 May 2024. This timeframe ensured that manual cleaning processes were observed under typical working conditions to accommodate for any variations in workload, technician skill levels, and different types of reusable flexible endoscopes. Not every reusable flexible endoscope manually cleaned during this period was directly observed, as a complete capturing of all manually cleaned reusable flexible endoscopes would have been impractical. Instead, a diverse and representative sample was included to reflect standard practices.

Observations did not extend beyond the core operational hours of the EDU, meaning out-of-hours manual cleaning procedures were not captured. However, as the majority of manual cleaning occurred within standard working hours, the data collected was expected to be representative of routine practice.

### 2.4. Data Collection

We used a DOTS as the data collection tool, a work measurement technique used to record the times and rates of working for the elements of a specified task carried out under defined conditions. The collected data were then analysed to determine the time necessary to perform the task at a defined level of performance. The observer applied a rating system to normalise the recorded times, ensuring consistency and accuracy in the time assessment [28].

A DOTS follows the principles of time and motion analysis, where an observer systematically records each action performed by a healthcare professional, along with the precise duration of each action. This approach accurately captures the length, frequency, and timing of activities, as well as the order of events, providing detailed insights into workflow patterns. By avoiding recall bias and subjective reporting, this method ensures a high reliability in identifying inefficiencies and establishing benchmarks for performance improvement. In hospitals and clinics, DOTSs are frequently used to evaluate the efficiency and time management of pharmacists, doctors, and nurses [29].

#### 2.4.1. Phase 1: Define the Manual Cleaning Process Using the Standard Detergent

During Week 1 of the study (12–15 and 18 March 2024), observers conducted daily observations between 9:00 a.m. and 5:00 p.m. over five days using the DOTS to define the manual cleaning process followed by decontamination technicians while using the standard detergent for the manual cleaning of reusable flexible endoscopes.

#### 2.4.2. Phase 2: Training, Familiarisation with, and Introduction of the New Detergent

To minimise the effects of direct observation, technicians were informed about the study’s purpose beforehand and allowed a one-week adjustment period, from 13 May to 19 May 2024, to familiarise themselves with the new detergent. Formal observations took place from 20 May to 24 May 2024. Observations were conducted in the same working environment to ensure that technicians performed their tasks under normal conditions, thereby reducing potential observer influence.

During the familiarisation period from 13 May to 19 May 2024, the new detergent replaced the standard one for the manual cleaning of reusable flexible endoscopes. According to its IFU, the new detergent allowed for the omission of the final rinse after manual cleaning, provided that the reusable flexible endoscopes were immediately reprocessed in a validated EWD [26,27].

Throughout this period, all decontamination technicians in the EDU underwent manufacturer-led training, which covered the correct detergent dosage—5 mL per litre of water for the new detergent, compared to 4 mL per litre for the standard detergent—and the omission of the final rinse after manual cleaning. Technicians received hands-on demonstrations and were required to demonstrate proficiency before independently performing manual cleaning.

#### 2.4.3. Phase 3: Define the Manual Cleaning Process Using the New Detergent

In Week 2 of the study, from 20 May to 24 May 2024, observers conducted daily observations between 9:00 a.m. and 5:00 p.m. over five days using DOTS to define the manual cleaning process with the new detergent.

Observations took place under standardised working conditions within the EDU to ensure consistency and maintain comparability between study phases. While some variability is inherent in real-world settings, efforts were made to minimise differences in workload, technician practices, and environmental factors such as staffing levels.

This phase was part of a pre-and post-intervention comparison designed to evaluate the impact of the new detergent on manual cleaning time, cleaning efficiency, and resource utilisation, compared to the standard detergent.

The study site adhered to its existing Standard Operating Procedure (SOP) for manual cleaning throughout the study. The only procedural change occurred in Phase 3, during which the final rinse after manual cleaning was omitted in accordance with the new detergent’s IFU.

#### 2.4.4. Measuring Decontamination Technicians’ Experience

Two surveys were conducted: one before the introduction of the new detergent (in Week 1 of the study) and one after its introduction (in Week 2 of the study). All surveys were anonymised, and responses were aggregated. The surveys were developed based on input from decontamination specialists and aimed to understand the perceived impact of the manual cleaning process before and after the introduction of the new detergent on decontamination technicians’ job satisfaction, environmental concerns, and workload. A pilot test was conducted internally with the study sponsor to refine the questions for clarity and usability.

### 2.5. Analysis

We utilised the DOTS as our data collection tool to record the time and work rate for specific tasks. This approach allowed us to analyse the time required to complete a task at a particular performance level.

All observations were manually entered into a secure tablet-based software package, SmartTime3 (ST3, build version 3.1.20190123), and were subsequently exported to Excel for analysis.

#### 2.5.1. Standard Minute Values

To evaluate the efficiency of the manual cleaning steps within the overall cleaning process, Standard Minute Values (SMVs) were used to compare the new and standard detergent workflows. SMVs quantify the total time required to complete a task under standard performance conditions, incorporating allowances for rest, fatigue, and minor disruptions. This approach ensures that the reported times reflect achievable and repeatable outcomes under typical working conditions [30].

SMVs were calculated by first recording the basic time taken for each task in the manual cleaning process based on direct observations of decontamination technicians using both detergents. These basic times were then adjusted using bundle allowances, which account for real-world factors that influence task duration and ensure consistency across observations.

Two bundle allowances were applied:

1. Relaxation Allowance (RA): Accounts for fatigue and short pauses during tasks, following established time study principles to reflect sustainable performance across longer shifts.
2. Contingency Allowance (CA): Captures unplanned variations in the cleaning process, such as minor workflow interruptions, additional scrubbing due to visible debris, and variation in endoscope complexity. It also accounts for any residual impact of the observer effect, which was minimised through familiarisation and routine workflow monitoring.

By incorporating these allowances, SMVs provided a structured and objective measure of manual cleaning efficiency. This approach ensured that the observed differences between the standard and new detergent processes—specifically, the removal of the final rinse step after manual cleaning—reflected true process changes rather than variations in individual task execution.

Due to data privacy constraints, individual task timing data were not retained. Instead, observations for each manual cleaning task were aggregated into total basic minutes and divided by the number of occurrences (i.e., the number of times each task was observed during the study period) to determine the average basic time per task. An RA and a CA were then applied to each average to produce the SMV per task occurrence. The SMVs for all tasks were then summed to calculate the total manual cleaning time. As a result, standard deviations and medians could not be calculated, and statistical tests for significance were not feasible. SMVs were therefore used as a consistent and repeatable method of process efficiency, accounting for real-world variability.

SMVs were selected over average or median times because they provide an objective benchmark that incorporates performance allowances. In contrast, average times can be skewed by outliers (e.g., one particularly slow or fast observation), while median times do not account for performance allowances. SMVs are reported in minutes and seconds to enable precise, direct comparisons between detergent workflows.

#### 2.5.2. Resource Cost Analysis

Following data collection, we conducted a resource cost analysis to estimate the average cost of manually cleaning one reusable flexible endoscope. This analysis aimed to project the potential cost savings for the EDU.

We developed a model that outlined the costs associated with detergents, water, consumables (such as disposable lint-free cloths, single-use channel brushes, and disinfectant wipes), and personnel costs. It is important to note that manufacturer-led training was provided at no additional cost and was therefore excluded from the cost analysis.

To estimate personnel costs, we used an average hourly rate based on data from the Unit Cost of Health and Social Care [31] as well as resource cost information provided by Blackpool Teaching Hospitals NHS Foundation Trust.

### 2.6. Ethics Approval and Consent

We completed the NHS Research Ethics Committee (REC) review tool available at https://www.hra-decisiontools.org.uk/ethics (accessed on 1 December 2023), which indicated that an NHS REC review was not required for this study. This research did not involve recording any patient, patient-related, or personal data from the decontamination technicians. Therefore, obtaining informed consent from patients was not applicable.

To ensure confidentiality and anonymity, no personally identifiable information was collected from the decontamination technicians. All survey responses were anonymised and aggregated, ensuring individual participants could not be identified in the analysis or reporting.

Prior to data collection, we consulted with all decontamination technicians to address any questions they had. The management team of Blackpool Victoria Hospital’s EDU provided written informed consent for conducting and publishing the study, as well as for conducting observations in the EDU, on behalf of all participants.

## 3. Results

### 3.1. Manual Cleaning Time

The DOTS identified that the EDU manually cleaned a variety of reusable flexible endoscopes, encompassing different makes, models, and types. Due to time constraints during the initial observation, the specific type of reusable flexible endoscope being cleaned was not recorded. Instead, tracking numbers were noted, which allowed for later categorisation of the reusable flexible endoscopes based on their characteristics and type.

Table 1 presents the subcategories and types of reusable flexible endoscopes alongside a comparison of the average manual cleaning times using the new detergent compared to the standard detergent. The time saved is expressed in both minutes and percentage reductions.

The results indicate that the new detergent consistently reduced manual cleaning times, as reflected in the lower SMVs, across all subcategories of reusable flexible endoscopes. The most significant reduction was observed in category C2 (channelled flexible endoscopes), with an average SMV reduction of 3 min and 55 s (28%). In category A3 (channelled flexible endoscopes), the average SMV reduction was 3 min and 15 s (22%). The smallest reduction occurred in category B (non-channelled flexible endoscopes), with an average SMV reduction of 41 s (10%).

To calculate the weighted average SMV, which reflects the manual cleaning time and considers the distribution of the manually cleaned reusable flexible endoscopes, we analysed the number of manually cleaned reusable flexible endoscopes in each subcategory from 1 April 2023 to 31 March 2024 (refer to Table 2). This calculation involved multiplying the SMV for each subcategory (as shown in Table 1) by the number of reusable flexible endoscopes cleaned within that subcategory. The resulting values were then summed and divided by the total number of manually cleaned reusable flexible endoscopes across all subcategories during the specified period.

This method ensured that the weighted average SMV accurately represented both manual cleaning time and the actual distribution of manually cleaned reusable flexible endoscopes, providing a reliable measure of overall cleaning efficiency.

As indicated in Table 3, the weighted average SMV of the manual cleaning time per reusable flexible endoscope, adjusted for reusable flexible endoscope distribution, was 11 min and 10.7 s with the new detergent, compared to 13 min and 10.2 s with the standard detergent. This resulted in an SMV reduction of 1 min and 59.5 s, which was a decrease of 15% (Table 4).

### 3.2. Utilisation Analysis

The term ‘utilisation’ refers to the EDU’s capacity to manually clean reusable flexible endoscopes in relation to the time required for decontamination technicians to perform this task. It reflects how efficiently the EDU utilises its resources to manually clean the throughput of these reusable flexible endoscopes.

#### 3.2.1. Current Utilisation (April 2023 to March 2024)

The EDU has a weekly allocation of 112.5 h (or 6750 min) for manual cleaning, which totals 351,000 min annually, based on the availability of decontamination technicians.

From April 2023 to March 2024, the EDU manually cleaned 24,217 reusable flexible endoscopes out of a total capacity of 26,652. The weighted average SMV for manual cleaning with the standard detergent was 13 min and 10.2 s per endoscope (see Table 4). This resulted in a utilisation rate of 91%, indicating a shortfall of 9% below capacity. This suggests that there is limited flexibility to accommodate increased demand (see Table A1).

When using the new detergent, the weighted average SMV for manual cleaning decreased to 11 min and 10.7 s per endoscope (see Table 4), leading to a projected utilisation rate of 77%, which is 23% below capacity (see Table A1).

#### 3.2.2. Projected Utilisation (April 2024 to March 2025)

The EDU anticipates the manual cleaning of a total of 29,000 reusable flexible endoscopes between April 2024 and March 2025. This represents an increase of 4783 endoscopes (20%) compared to the same period in the previous year.

Using the standard detergent, the projected utilisation rate is expected to be 109%, which would exceed capacity by 9% (see Table A1). In contrast, with the new detergent, the projected utilisation rate is estimated to be 92%, staying within the acceptable limits at 8% below capacity (see Table A1).

#### 3.2.3. Time Requirements for Decontamination Technicians

The manual cleaning process using the standard detergent required approximately 122.41 h per week from the decontamination technicians. With the introduction of the new detergent, this requirement was reduced to about 103.89 h per week, resulting in a weekly saving of approximately 18.52 h. Over the course of one year, this reduction translated to an estimated total savings of around 962.9 h for decontamination technicians (see Table A1). These findings indicate that the new detergent enables the same volume of reusable flexible endoscopes to be manually cleaned while optimising personnel resources within the EDU.

### 3.3. Water Usage During Manual Cleaning

When using the standard detergent, decontamination technicians filled two sinks to the designated fill line, each containing 25 L of water, leading to a total of 50 L. The reusable flexible endoscope was first submerged in Sink 1 with the standard detergent and was subsequently flushed. It was then transferred to Sink 2 for rinsing in fresh water. A detailed description of this process is available in Figure S1.

With the introduction of the new detergent, the need for Sink 2 has been eliminated, as the final rinse after manual cleaning can be omitted when using a validated EWD. This change saves 25 L of water per manually cleaned reusable flexible endoscope. Blackpool Victoria Hospital’s EDU anticipates the manual cleaning of 29,000 reusable flexible endoscopes between March 2024 and April 2025, resulting in estimated annual water savings of 725,000 litres when using the new detergent compared to the standard detergent (725,000 litres vs. 1,450,000 litres).

### 3.4. Resource Cost Analysis

While most consumables remained consistent between the two cleaning methods, notable differences were observed in detergent and water usage as well as personnel time due to variations in manual cleaning efficiency.

The analysis revealed that the total cost per manually cleaned reusable flexible endoscope was slightly higher with the standard detergent at GBP 4.90 (see Table 5) compared to GBP 4.88 with the new detergent (see Table 6), representing a reduction of 0.41%. This difference translated to estimated annual savings of GBP 606 *, based on the projected manual cleaning of 29,000 reusable flexible endoscopes from March 2024 to April 2025 when using the new detergent. The comparative costs of manually cleaning reusable flexible endoscopes using standard and new detergents are found in Table 7.

* Figures for the “total cost per manually cleaned reusable flexible endoscope” (see Table 5 and Table 6) have been rounded to two decimal places for clarity. As a result, the total annual savings of GBP 606 slightly differ from the simplified calculation of GBP 0.02 (GBP 4.88 vs. GBP 4.90) per endoscope multiplied by 29,000 endoscopes.

### 3.5. Decontamination Technicians’ Experience

#### 3.5.1. Decontamination Technicians’ Experience of Using the Standard Detergent

All decontamination technicians involved in the manual cleaning of reusable flexible endoscopes were invited to participate in a survey (see Figure 1). Out of 12 technicians, 11 responded, resulting in a response rate of 92%.

On average, the respondents had been using the standard detergent and working in the EDU for a total of 26 months and 4 days, which reflected their average length of employment in the unit.

#### 3.5.2. Decontamination Technicians’ Experience of Using the New Detergent

**Figure 2:**
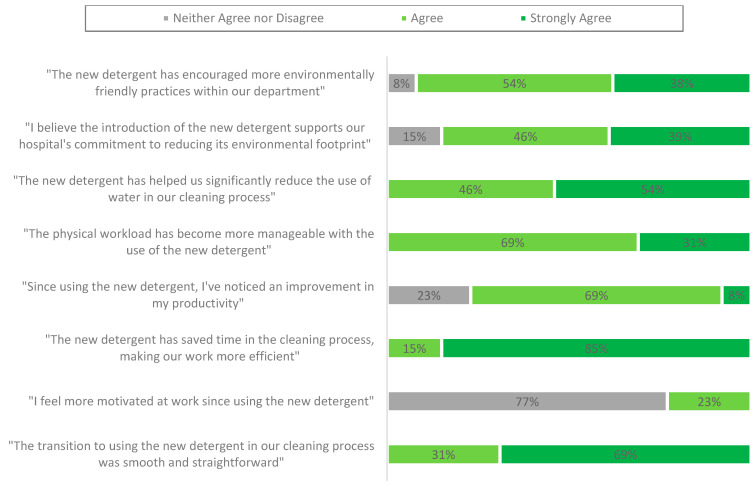
Impact of the new detergent on decontamination technician experiences.

After the implementation of the new detergent for the manual cleaning of reusable flexible endoscopes, another survey was conducted among 14 decontamination technicians who were invited to participate, with 13 responding. This resulted in a response rate of 93% (see Figure 2). The respondents had been using the new detergent for 10 working days, from 13 May to 24 May 2024.

## 4. Discussion

### 4.1. Water Consumption and Environmental Impact

This study demonstrates that adopting the new detergent supports NHS sustainability targets and addresses the increasing pressures on healthcare resources by improving efficiency and reducing costs. Specifically, the new detergent allows for the elimination of the final rinse after the manual cleaning of reusable flexible endoscopes, which is a key part of the reprocessing cycle. This innovation enables NHS facilities to reduce water usage, enhance technician productivity, and lower their environmental footprints.

We estimate an annual reduction in water consumption of approximately 725,000 litres as a result of this intervention. This aligns directly with NHS goals to achieve net-zero emissions by 2040 and to reduce carbon emissions by 80% between 2028 and 2032 [2]. These water savings are not only environmentally significant but also reduce the carbon footprint associated with water treatment and supply [32]. Furthermore, NHS’s sustainability targets emphasise optimising water use to align with climate goals amid growing concerns about water scarcity in densely populated areas [33,34].

Reusable flexible endoscope reprocessing is known to be resource-intensive [10,11]. Research indicates that each complete reprocessing cycle for a reusable flexible endoscope uses between 55 and 113 L of water, depending on local protocols and equipment [11,35,36]. Manual cleaning alone is estimated to account for 15–20 L of that total [11,35,36]. Some broader environmental audits report even higher total water usage per reprocessing cycle, ranging from 80 to 114 L, although these figures often do not distinguish between the specific steps of the process (e.g., pre-cleaning, manual cleaning, disinfection, rinsing, drying) [37].

Given that approximately 1.73 million endoscopic procedures were performed in the NHS in 2023–2024, improving the efficiency of the reprocessing workflow—especially during manual cleaning—represents a significant opportunity to reduce healthcare’s environmental burden [38,39].

The European Society of Gastrointestinal Endoscopy (ESGE) recommends minimising water use in endoscopy reprocessing [40]. Similarly, the British Society of Gastroenterology (BSG) and the Centre for Sustainable Healthcare (CSH) have issued joint consensus statements promoting environmental sustainability in endoscopy. Their guidance specifically recommends that units implement an SOP to rationalise and reduce water consumption wherever possible [37].

Efficient water use in UK hospitals is essential for both operational and environmental sustainability. NHS hospitals currently consume large volumes of water for the reprocessing of reusable flexible endoscopes, contributing significantly to the sector’s overall environmental impact [41,42].

When using the standard detergent, decontamination technicians filled two sinks simultaneously to the designated fill line, each containing 25 L of water, resulting in a total of 50 L. The reusable flexible endoscope was first submerged in Sink 1 with the standard detergent and then flushed. It was subsequently transferred to Sink 2 for rinsing in fresh water. However, with the new detergent, the need for Sink 2 was eliminated, as the final rinse after manual cleaning could be omitted when using a validated EWD. This change saved 25 L of water per manually cleaned reusable flexible endoscope.

Reducing water consumption by 25 L per manually cleaned reusable flexible endoscope, as demonstrated in this study, represents a measurable and replicable step toward greener healthcare practices. This research also highlights how the selection of detergent type can influence water usage in manual cleaning protocols.

Published environmental assessments indicate that a reusable flexible endoscope reprocessing cycle may consume as much as 22–30 gallons (approximately 83–114 L) of water [43,44]. While much of this volume is attributable to automated disinfection, our findings show that the manual cleaning stage alone contributes a substantial share and is a practical target for water-saving interventions.

### 4.2. Cost Savings and Efficiency Gains

Our study demonstrates that the per-unit cost reduction for manually cleaning reusable flexible endoscopes is marginal, decreasing from GBP 4.90 to GBP 4.88. However, when scaled to 29,000 manually cleaned reusable flexible endoscopes, this results in a cumulative annual savings of GBP 606.

These findings contrast with the previous literature, which reported the cost of manually cleaning a reusable flexible endoscope to range from GBP 8.60 to GBP 28.73. These figures included both materials and technician time [24]. Furthermore, one UK-based analysis reported the cost of reprocessing a reusable flexible endoscope as GBP 45 [44,45]. However, this figure did not distinguish between the specific steps involved in the cleaning process (e.g., pre-cleaning, manual cleaning, disinfection, rinsing, drying).

The discrepancies between our findings and those reported in the existing literature may be attributed to variations in workflow efficiency, study design, assumptions in cost modelling, and the types of cleaning materials used.

Importantly, the primary financial benefit stems not solely from a lower detergent cost, but from an improvement in cleaning efficiency. As indicated in Table 4, the weighted average SMV, adjusted for reusable flexible endoscope distribution, was reduced from 13 min and 10.2 s with the standard detergent to 11 min and 10.7 s with the new detergent. This represented a time saving of 1 min and 59.5 s, or approximately 15%. This reduction in time translated to higher throughput, as technicians could now manually clean 5.401 reusable flexible endoscopes per hour compared to 4.556 with the standard detergent. Consequently, this enhanced efficiency reduced labour costs per reusable flexible endoscope, which is crucial in high-throughput settings.

The reduction in cleaning time—equating to a 15% decrease in SMV—appeared to be the main driver of cost efficiency. This improvement outweighed the modest increase in detergent cost (GBP 1.08 compared to GBP 0.59 per reusable flexible endoscope) and the slight reduction in water costs (GBP 0.03 compared to GBP 0.07). This indicates that even modest improvements in manual cleaning speed and resource utilisation can lead to meaningful operational savings, particularly in high-throughput environments where labour costs constitute a large proportion of the total expenses.

Additionally, this decrease in the SMV had a tangible impact on staffing requirements. The manual cleaning process using the standard detergent required approximately 122.41 technician hours per week. With the new detergent, this requirement dropped to about 103.89 h per week, representing a weekly savings of approximately 18.52 h. Extrapolated over one year, this efficiency gain resulted in an estimated savings of around 962.9 technician hours (see Table A1). These findings suggested that the new detergent not only supported the cleaning of the same volume of reusable flexible endoscopes but also optimised personnel resources within the EDU.

Overall, while the per-scope cost savings may seem modest, the cumulative impact, when coupled with increased operational efficiency, presents a compelling case for the adoption of the new detergent. The efficiency gains not only lower labour costs but also improve overall workflow, allowing healthcare facilities to reallocate savings towards other critical areas, such as staff training.

### 4.3. Enhanced Productivity and Staff Awareness:

- NHS water management practices involve upgrading equipment to minimise water waste and raising staff awareness about efficient water use [7,33,46]. Our survey revealed that 77% of respondents reported improved productivity with the new detergent, while 100% acknowledged a more manageable workload. This increased efficiency allowed technicians to focus on other critical tasks. All respondents agreed or strongly agreed that the new detergent significantly reduced water usage during the manual cleaning process and contributed to time savings, thus enhancing the efficiency of decontamination technicians.
- Interestingly, 77% of respondents selected ‘neither agree nor disagree’ when asked if they felt more motivated at work since using the new detergent. This may have indicated that while the change improved workflow efficiency, it had a limited impact on perceived job satisfaction or motivation. This neutrality could be attributed to role constraints, the indirect nature of the intervention, or the timing of the post-survey, which occurred shortly after training.

### 4.4. Limitations

While this study offered valuable insights into the benefits of using a novel neutral detergent, several limitations should be acknowledged.

First, the study was conducted at a single NHS facility over a short duration. Although observations spanned multiple shifts and technician teams, the findings may not be generalisable to other settings due to differences in workflows, training, infrastructure, and local policies. To address this limitation, multi-centre evaluations over extended periods are necessary.

Second, raw task-level data were unavailable for analysis or sharing due to data privacy constraints. The time study data were provided in an aggregated format, which prevented the use of standard statistical tests (e.g., standard deviations, confidence intervals, or *p*-values). To mitigate this, SMVs were used, which incorporated structured allowances to account for workflow variability. Future research should prioritise access to individual-level time data to enable inferential statistical testing.

Third, the descriptive nature of this study precluded causal inferences. Further experimental research is needed to establish causal relationships between the intervention and observed outcomes.

Fourth, formal inter-rater reliability testing was not conducted. While observers followed a structured handover protocol and were trained to similar standards in work measurements to maintain consistency, minor variations in task classification may have occurred. Future studies should incorporate formal reliability testing to enhance rigour.

Fifth, while reusable flexible endoscopes were categorised into three types—channelled, non-channelled, and specialist—the complexity of manual cleaning across individual models was not examined in detail. The observed variation in SMV reduction across scope categories suggests that future research should explore these nuances, incorporating scope-level analysis by make, model, and complexity.

Sixth, this study did not assess compliance with the IFU regarding reprocessing. The recorded manual cleaning times reflected the local hospital protocols at the study site and may not have represented the time required to complete all steps as outlined in the IFU. Future studies should assess compliance with IFU-recommended reprocessing steps.

Seventh, feedback from decontamination technicians was gathered through pre- and post-intervention surveys designed for qualitative insight, but without external validation. Although these surveys were internally piloted, broader pre-testing was not conducted, and the responses may have reflected perception rather than objective performance data. The sample may also not have represented the full range of technician experiences. Future studies should employ validated survey instruments and larger, more representative samples.

## 5. Conclusions

The new detergent demonstrates measurable improvements in water conservation, operational efficiency, and resource costs, supporting the NHS’s sustainability goals. Scaling this solution across healthcare facilities could significantly contribute to achieving net-zero targets while tackling operational challenges in reusable flexible endoscope reprocessing.

While the findings of this study indicate meaningful improvements in water conservation and process efficiency, the inability to perform statistical testing and the limited generalisability remain important limitations. Nonetheless, the methodology used provides a structured assessment of operational impact in a high-volume NHS setting. Future research should build upon this by utilising raw task-level datasets and multi-site evaluations to validate these findings and inform broader adoption within the NHS.

## Figures and Tables

**Table 1:** Comparison of average SMVs for manual cleaning of reusable flexible endoscopes using the new and standard detergents.

Sub-Category	Type of Reusable Flexible Endoscope	Average SMV Per Flexible Endoscope (New Detergent) (min, s)	Average SMV Per Flexible Endoscope (Standard Detergent) (min, s)	SMV Reduction with New Detergent (min, s)	SMV Reduction (%)
A1	Colonoscope, Gastroscope, Paediatric	12 min, 42.4 s	15 min, 12.7 s	2 min, 30 s	16%
A2	Urology Scopes (Cystoscope)	10 min, 55.8 s	12 min, 17.2 s	1 min, 21 s	11%
A3	Inpatient (Ureteroscope)	11 min, 13.5 s	14 min, 28.6 s	3 min, 15 s	22%
B	ENT, Cardiac Probes	6 min, 4.8 s	6 min, 46.0 s	41 s	10%
C1	Therapeutic/Echo Scopes	14 min, 30.2 s	18 min, 30.3 s	4 min	22%
C2	Pulmonary and Ultrasound Scopes	10 min, 16.2 s	14 min, 10.4 s	3 min, 55 s	28%
C3	Video Bronchoscope	9 min, 20.4 s	11 min, 51.2 s	2 min, 31 s	21%

Note: The ‘Average SMV Per Flexible Endoscope’ columns indicate the SMV required to manually clean a reusable flexible endoscope using the new or the standard detergent during manual cleaning. SMVs account for standard allowances, including relaxation and contingency, ensuring a structured and repeatable measure of the manual cleaning time. The ‘SMV Reduction with New Detergent’ represents the reduction in SMV achieved by using the new detergent instead of the incumbent detergent during manual cleaning. Reusable flexible endoscopes are categorised as channelled flexible endoscopes (A1, A2, A3), non-channelled flexible endoscopes (B), or specialist flexible endoscopes (C1, C2, C3) based on their design and intended use. All percentages have been rounded to the nearest whole number for simplicity. All time values are rounded to one decimal place for precision.

**Table 2:** Distribution of manually cleaned reusable flexible endoscopes by subcategory from 1 April 2023 to 31 March 2024.

Subcategories	Flexible Endoscopes Manually Cleaned from 1 April 2023 to 31 March 2024	Percentage of Total (%)
A1	15,174	63%
A2	4253	18%
A3	346	1%
B	4351	18%
C	92	<1%
Total	24,217	100%

Note: The table presents the distribution of the manually cleaned reusable flexible endoscopes across subcategories from 1 April 2023 to 31 March 2024. The total number of manually cleaned reusable flexible endoscopes is 24,217, and the percentages indicate each subcategory’s contribution to the overall cleaning volume. Percentages are rounded to the nearest whole number, with values below 1% shown as ‘<1%’. The subcategories are defined as follows: A1: Colonoscope, Gastroscope, Paediatric Endoscope; A2: Urology Scopes (e.g., Cystoscope); A3: Inpatient Endoscopes (e.g., Ureteroscope); B: Non-channelled Endoscopes (e.g., ENT, Cardiac Probes); C: Specialist Endoscopes (e.g., Bronchoscopes, Flexible Ultrasound Scopes).

**Table 3:** Comparison of average SMVs for manual cleaning time per reusable flexible endoscope using the new and the standard detergents.

SMV Per Flexible Endoscope (New Detergent) (min, s)	SMV Per Flexible Endoscope (Standard Detergent) (min, s)	SMV Reduction with New Detergent (min, s)	SMV Reduction (%)
11 min, 10.7 s	13 min, 10.2 s	1 min, 59.47 s	15%

Note: SMVs represent manual cleaning times under standardised conditions, incorporating allowances for relaxation and contingencies to ensure a structured, repeatable measure of efficiency. All SMVs are reported in minutes and seconds for accuracy. Percentages have been rounded to the nearest whole number, and all-time values are rounded to the nearest second.

**Table 4:** Comparison of weighted SMVs between the new and the standard detergents across reusable flexible endoscope categories.

Category	Weighted Average SMV (New Detergent) (min, s)	Weighted Average SMV (Standard Detergent) (min, s)	SMV Reduction with the New Detergent (min, s)	SMV Reduction (%)
A	12 min, 17.9 s	14 min, 34.2 s	2 min, 6.0 s	16%
B	6 min, 4.8 s	6 min, 46.0 s	41.0 s	10%
C	12 min, 22.3 s	14 min, 50.6 s	3 min, 28.0 s	23%

Note: All percentages have been rounded to the nearest whole number for simplicity. All SMVs are reported in minutes and seconds for accuracy. Percentages have been rounded to the nearest whole number for simplicity, and all-time values are rounded to the nearest second.

**Table 5:** Cost breakdown of manually cleaning reusable flexible endoscopes using the standard detergent.

Cleaning Materials	Unit Price (GBP)	Quantity Used per Flexible Endoscope	Cost per Manually Cleaned Flexible Endoscope (GBP) ^3^	Total Annual Cost (GBP) ^2^
Detergent (10 L)	GBP 59.00	100 mL	GBP 0.59	GBP 17,110
Water (50 L)	GBP 0.07	50 L	GBP 0.07	GBP 2001
Disposable lint-free cloth	GBP 94.28	1 per flexible endoscope	GBP 0.12	GBP 3418
Single-use channel brush	GBP 99.00	1 per flexible endoscope	GBP 0.99	GBP 18,710
Disinfectant wipes for sinks and counters	GBP 21.49	1 per flexible endoscope	GBP 0.11	GBP 3116
**Total material costs (GBP):**			GBP 1.87	GBP 54,355
**Personnel costs (GBP): ^1^**			GBP 3.03	GBP 87,841
Total cost per manually cleaned reusable flexible endoscope (GBP): *			GBP 4.90	GBP 142,196

^1^ Personnel costs are based on an hourly rate of GBP 11.50, plus a 20% on-cost [31], calculated at a cleaning rate of 4.556 endoscopes per hour. ^2^ Annual costs are calculated based on the projection of 29,000 endoscopes cleaned per year. ^3^ All cost figures have been rounded to two decimal places for clarity.

**Table 6:** Cost breakdown of manually cleaning reusable flexible endoscopes using the new detergent.

Cleaning Materials	Unit Price (GBP)	Quantity Used per Flexible Endoscope	Cost per Manually Cleaned Flexible Endoscope (GBP) ^3^	Total Annual Cost (GBP) ^2^
Detergent (10 L)	GBP 86.24	125 mL	GBP 1.08	GBP 31,262
Water (25 L)	GBP 0.07	25 L	GBP 0.03	GBP 1001
Disposable lint-free cloth	GBP 94.28	1 per flexible endoscope	GBP 0.12	GBP 3418
Single-use channel brush	GBP 99.00	1 per endoscope	GBP 0.99	GBP 28,710
**Disinfectant wipes for sinks and counters**	GBP 21.49	1 per flexible endoscope	GBP 0.11	GBP 3116
**Total material costs (GBP):**			GBP 2.33	GBP 67,506
**Personnel costs (GBP): ^1^**			GBP 2.55	GBP 74,083
Total cost per manually cleaned reusable flexible endoscope (GBP): *			GBP 4.88	GBP 141,590

^1^ Personnel costs are based on an hourly rate of GBP 11.50, plus a 20% on-cost [31], calculated at a cleaning rate of 5.402 endoscopes per hour. ^2^ Annual costs are calculated based on the projection of 29,000 endoscopes cleaned per year. ^3^ All cost figures have been rounded to two decimal places for clarity.

**Table 7:** Comparative costs of manually cleaning reusable flexible endoscopes using standard and new detergents.

	Standard Detergent (GBP)	New Detergent (GBP)
**Total Cost per Flexible Endoscope**	GBP 4.90	GBP 4.88
**Total Annual Cost (29,000 Endoscopes)**	GBP 142,196	GBP 141,590

## Data Availability

The data presented in this study are available on request from the corresponding author due to commercial restrictions.

## Supplementary Materials

The following supporting information can be downloaded at: https://www.mdpi.com/article/10.3390/jmahp13020023/s1, Figure S1: Endoscopy Decontamination Unit process maps.

## Appendix A

**Table A1:** Comparison of utilisation and manual cleaning capacity for the standard and the new detergents.

**Metric**	**Standard Detergent**	**New Detergent**
Weekly Hours Allocated	112.5	112.5
Weekly Minutes Allocated	6750	6750
Annual Minutes Allocated	351,000	351,000
Weighted Average Scope Manual Cleaning Time (minutes, seconds)	13 min, 10.2 s	11 min, 10.7 s
Manually Cleaned Reusable Flexible Endoscopes (April 2023 to March 2024)	24,217	24,217
Current Reusable Flexible Endoscope Manual Cleaning Capacity	26,652	26,652
Utilisation (%)	90.86%	77.12%
Forecasted Reusable Flexible Endoscopes (March 2024 to April 2025)	29,000	29,000
Forecasted Utilisation (March 2024 to April 2025) (%)	108.81%	92.35%

Note: The calculation of decontamination technicians’ time savings is based on the total weekly time required for manual cleaning using the standard and the new detergents. Cleaning with the standard detergent required 122.41 h per week, whereas the new detergent required 103.89 h per week, resulting in a weekly time saving of 18.52 h. Over the course of a year, this amounts to an estimated total time saving of approximately 962.9 h for decontamination technicians.
