# Supplementary material for: Streamlining Endoscopy Cleaning: The Impact of a New Detergent on Time and Water Use
Source: J Mark Access Health Policy. 2025 May 16;13(2):23. doi: 10.3390/jmahp13020023 (PMC12101361; doi:10.3390/jmahp13020023)
Supplement: Supplementary file 1 [file jmahp-13-00023-s001.zip › jmahp-3475535-supplementary.pdf]

Scope Manual Cleaning Process

SC1.0 Prepare for Cleaning Cycle and Get Scope

SC2.0 Clean Sub-Cat A1/ C1 Scope
Inc. Colon, Gas, PAED, Linear, Therapeutic, Radial, ERCP

SC2.1 Clean Sub-Cat A2/ C2 Scope
Inc. Urology, Bronch, EBUS

SC2.2 Clean Sub-Cat A3/ C3 Scope
Inc. In-Patient, Video Bronch

SC2.3 Clean Sub-Cat B Scope
Inc. ENT, Xion, Cardiac

SC3.0 Prepare Scope for Machine Cleaning

Scope Manual Cleaning Process Summary

- This is a summary of the scope cleaning process.
- This process map does not include all steps such as collection and receival of scopes, or machine cleaning of scopes and the subsequent processes involved in the full scope cleaning cycle.
- This process map does not include steps taken to process a defective scope, as the detergent used in the cleaning cycle will have no bearing.
- The process begins assuming a scope is present in the hatch.

Key Drivers:
Scopes

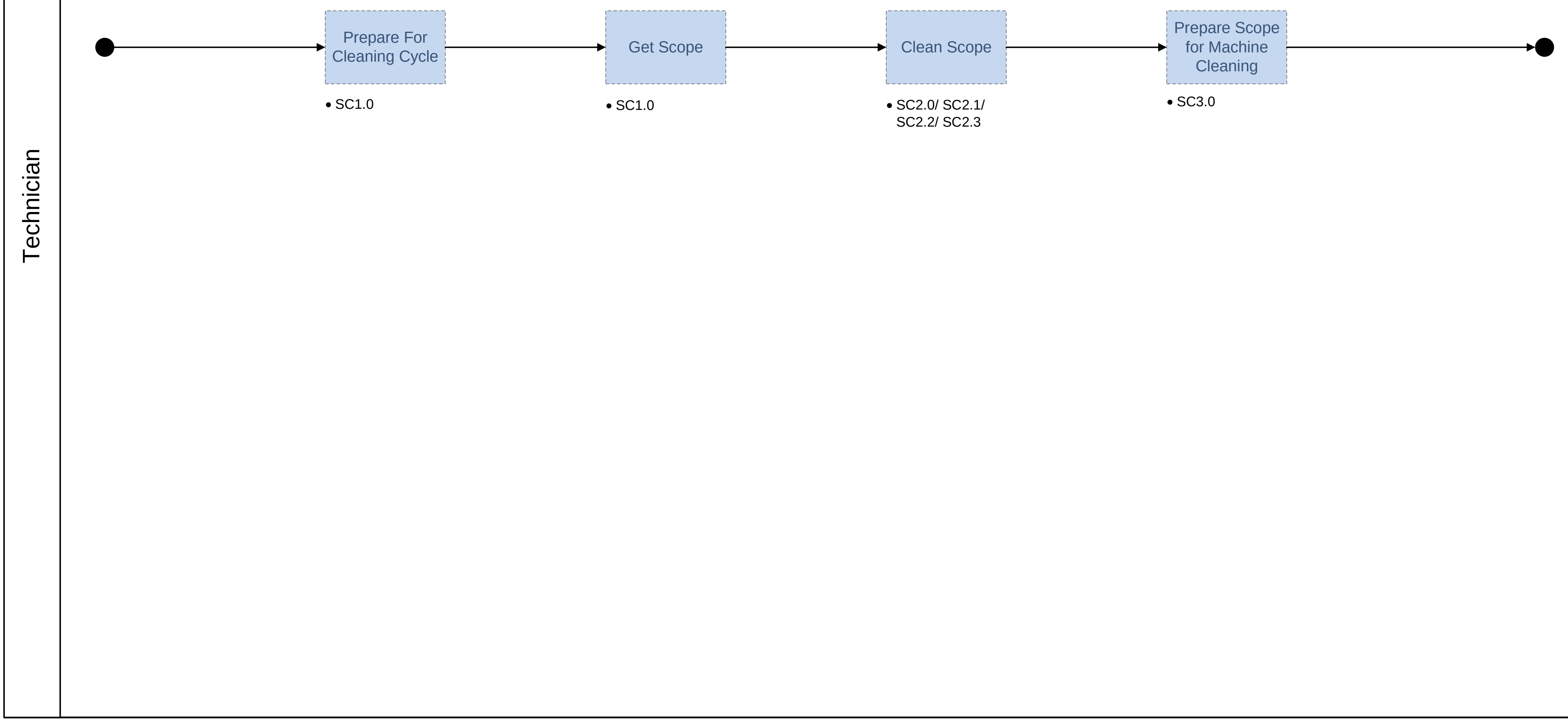

SC1.0 Prepare for Cleaning Cycle and Get Scope

Technician

- This is the process of preparing to carry out a manual cleaning cycle.
- This process map does not include all steps such as collection and receival of used scopes.
- The process begins assuming a scope is present in the hatch.

Key Drivers:
Endoscopes used

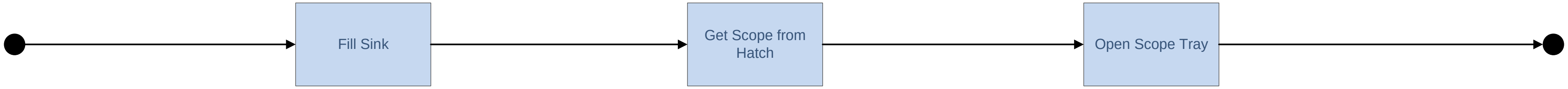

Fill Sink

- Both sinks should be filled at the same time.
- The sinks have mixer taps but these must be monitored or water temperature may not be correct.
- Temperature range is 27-35 degrees Celsius.
- Sinks must be filled to the -fill line-, which measures 25L of water.

Get Scope from Hatch

- Process assumes there is a scope requiring cleaning present in the hatch.
- Process assumes technicians are trained to clean the scope present in the hatch.

Open Scope Tray

- Scope trays are sealed with red plastic covers. On these covers information on scope type and receival time can be found.
- Red plastic covers are asided to medical waste bin after removal.

Freq:	Fill Sink	Get Scope from Hatch	Open Scope Tray
	Per scope	Per scope	Per scope

SC2.0 Clean Sub-Cat A1/ C1 Scope - Inc. Colon, Gas, PAED, Linear, Therapeutic, Radial, ERCP

Technician

- This process describes the process of machine leak testing, cleaning, and machine flushing a channelled or specialist scope (Colon, Gas, PAED, Linear, Therapeutic, Radial, ERCP). These scopes are categorised as A1 or C1 type scopes.
- All scopes must be leak tested prior to submersion to minimise damage to parts of the scope not designed for fluid exposure.
- The process assumes no leaks are found
- The process assumes all consumables are readily available.

Key Drivers:
Scopes

Connect Leak Test Hose

- Only applicable to scopes compatible with Scopetech, else refer to SC2.2 or SC2.3.
- Scopetech air out connection port to scope.
- Install waterproof cap on scope.
- Attach leak tester hose to venting connection on waterproof cap.
- More detailed instructions are available in department SOP’s.

Freq: Per scope

Machine Leak Test Scope

- Align pins on scope.
- Initiate leak test on Scopetech.
- Fill the scope with air before submerging in sink 1.
- Submerge scope in sink 1 and observe for escaping air.
- After approximately 30 seconds of observation, remove the scope from sink 1.
- More detailed instructions are available in department SOP’s.

Freq: Per scope

Disconnect Leak Test Hose

- Discontinue the leak test on Scopetech.
- Release air pressure by disconnecting leak test hose from Scopetech.
- Reconnect leak test hose to Scopetech.
- Disconnect leak test hose from scope and aside.
- More detailed instructions are available in department SOP’s.

Freq: Per scope

Add Detergent to Sink

- 100ml Neutrascope is dispensed to sink 1 when input on Scopetech.

Freq: Per scope

Clean Outside Surface of Scope

- Using consumables described in SOP’s, wipe clean the outer surfaces of the scope and accompanying components in sink 1 (with added detergent).
- More detailed cleaning instructions are available in department SOP’s.

Freq: Per scope

Pipe Clean Channels

- Using consumables described in SOP’s, clean any channels or hard to access areas of scope and accompanying components in sink 1 (with added detergent).
- More detailed cleaning instructions are available in department SOP’s.

Freq: Per scope

Connect Flush Lines to Scope

- Connect air/ water channel blocker.
- Connect suction valve tube to the suction port.
- Connect silicone flush tube to scope air pump connection.
- Attach water bottle connection blocker.
- Connect auxiliary water port to scope.
- More detailed flushing instructions are available in department SOP’s.

Machine Flush Scope Channels

- Submerge scope in sink 1 (with added detergent).
- Submerge flush pump suction tube in sink 1 (with added detergent).
- Initiate scope flushing on Scopetech.
- More detailed flushing instructions are available in department SOP’s.

Machine Rinse Scope Channels

- Transfer scope to sink 2 (with clean, fresh water) when machine flush is complete.
- Ensure scope is submerged.
- Submerge flush pump suction tube in sink 2 (with clean, fresh channels).
- Initiate scope rinsing on Scopetech.
- More detailed flushing instructions are available in department SOP’s.

Empty Sinks

- Remove sink “plugs” to start emptying of sinks.

Machine Purge Scope Channels

- Remove flush pump suction tube from sink.
- Initiate scope purging on Scopetech.
- More detailed flushing instructions are available in department SOP’s.

Disconnect Flush Lines from Scope

- Disconnect air/ water channel blocker.
- Disconnect suction valve tube from the suction port.
- Disconnect silicone flush tube from scope air pump connection.
- Detach water bottle connection blocker.
- Disconnect auxiliary water port from scope.
- More detailed cleaning instructions are available in department SOP’s.

SC2.1 Clean Sub-Cat A2/ C2 Scope - Inc. Urology, Bronch, EBUS

Technician

- This process describes the process of machine leak testing, cleaning, and manually flushing a channelled or specialist scope (Urology, Bronch, EBUS). These scopes are categorised as A2 or C2 type scopes.
- All scopes must be leak tested prior to submersion to minimise damage to parts of the scope not designed for fluid exposure.
- The process assumes no leaks are found
- The process assumes all consumables are readily available.

Key Drivers:
Scopes

Connect Leak Test Hose

- Only applicable to scopes compatible with Scopetech, else refer to SC2.2 or SC2.3.
- Scopetech air out connection port to scope.
- Install waterproof cap on scope.
- Attach leak tester hose to venting connection on waterproof cap.
- More detailed instructions are available in department SOP's.

Freq: Per scope

Machine Leak Test Scope

- Align pins on scope.
- Initiate leak test on Scopetech.
- Fill the scope with air before submerging in sink 1.
- Submerge scope in sink 1 and observe for escaping air.
- After approximately 30 seconds of observation, remove the scope from sink 1.
- More detailed instructions are available in department SOP's.

Disconnect Leak Test Hose

- Discontinue the leak test on Scopetech.
- Release air pressure by disconnecting leak test hose from Scopetech.
- Reconnect leak test hose to Scopetech.
- Disconnect leak test hose from scope and aside.
- More detailed instructions are available in department SOP's.

Add Detergent to Sink

- 100ml Neutrascope is dispensed to sink 1 when input on Scopetech.

Clean Outside Surface of Scope

- Using consumables described in SOP's, wipe clean the outer surfaces of the scope and accompanying components in sink 1 (with added detergent).
- More detailed cleaning instructions are available in department SOP's.

Pipe Clean Channels

- Using consumables described in SOP's, clean any channels or hard to access areas of scope and accompanying components in sink 1 (with added detergent).
- More detailed cleaning instructions are available in department SOP's.

Freq: Per scope

Manually Flush Scope Channels

- Submerge scope in sink 1 (with added detergent).
- Using a syringe, manually flush scope channels using water/ detergent mix from sink 1 (with added detergent).
- Repeat following department SOP's.
- More detailed flushing instructions are available in department SOP's.

Freq: Per scope

Manually Rinse Scope Channels

- Transfer scope to sink 2 (with clean, fresh water).
- Using a syringe, manually rinse scope channels using clean, fresh water from sink 2.
- Repeat following department SOP's.
- More detailed flushing instructions are available in department SOP's.

Freq: Per scope

Empty Sinks

- Remove sink "plugs" to start emptying of sinks.

Freq: Per scope

Manually Purge Scope Channels

- Using a syringe, manually purge scope channels using air.
- Repeat following department SOP's.
- More detailed flushing instructions are available in department SOP's.

Freq: Per scope

SC2.2 Clean Sub-Cat A3/ C3 Scope - Inc. In-Patient, Video Bronch

Technician

- This process describes the process of manually leak testing, cleaning, and manually flushing a channelled or specialist scope (In-Patient, Video Bronch). These scopes are categorised as A3 or C3 type scopes.
- All scopes must be leak tested prior to submersion to minimise damage to parts of the scope not designed for fluid exposure.
- The process assumes no leaks are found
- The process assumes all consumables are readily available.

Key Drivers:
Scopes

Manually Leak Test Scope

- Obtain handheld pump compatible with scope – several pumps are available in the brush room.
- Attach handheld pump to scope.
- Pump air in to scope until air pressure within scope has stabilised.
- Observe pressure needle on handheld pump until satisfied there is no leak (which would be indicated by a drop in pressure).
- Disconnect handheld pump from scope and return to storage location.
- More detailed instructions are available in department SOP's.

Add Detergent to Sink

- 100ml Neutrascope is dispensed to sink 1 when input on Scopetech.

Clean Outside Surface of Scope

- Using consumables described in SOP's, wipe clean the outer surfaces of the scope and accompanying components in sink 1 (with added detergent).
- More detailed cleaning instructions are available in department SOP's.

Pipe Clean Channels

- Using consumables described in SOP's, clean any channels or hard to access areas of scope and accompanying components in sink 1 (with added detergent).
- More detailed cleaning instructions are available in department SOP's.

Manually Flush Scope Channels

- Submerge scope in sink 1 (with added detergent).
- Using a syringe, manually flush scope channels using water/ detergent mix from sink 1 (with added detergent).
- Repeat following department SOP's.
- More detailed flushing instructions are available in department SOP's.

Manually Rinse Scope Channels

- Transfer scope to sink 2 (with clean, fresh water).
- Using a syringe, manually rinse scope channels using clean, fresh water from sink 2.
- Repeat following department SOP's.
- More detailed flushing instructions are available in department SOP's.

Empty Sinks

- Remove sink "plugs" to start emptying of sinks.

Manually Purge Scope Channels

- Using a syringe, manually purge scope channels using air.
- Repeat following department SOP's.
- More detailed flushing instructions are available in department SOP's.

Freq:				
	Per scope	Per scope	Per scope	Per scope

SC2.3 Clean Sub-Cat B Scope - Inc. ENT, Xion, Cardiac

Technician

- This process describes the process of manually leak testing and cleaning a non-channelled scope (ENT, Xion, Cardiac). These scopes are categorised as B type scopes.
- All scopes must be leak tested prior to submersion to minimise damage to parts of the scope not designed for fluid exposure.
- The process assumes no leaks are found
- The process assumes all consumables are readily available.

Key Drivers:
Scopes

Manually Leak Test Scope

- Obtain handheld pump compatible with scope – several pumps are available in the brush room.
- Attach handheld pump to scope.
- Pump air in to scope until air pressure within scope has stabilised.
- Observe pressure needle on handheld pump until satisfied there is no leak (which would be indicated by a drop in pressure).
- Disconnect handheld pump from scope and return to storage location.
- More detailed instructions are available in department SOP’s.

Add Detergent to Sink

- 100ml Neutrascope is dispensed to sink 1 when input on Scopetech.

Clean Outside Surface of Scope

- Using consumables described in SOP’s, wipe clean the outer surfaces of the scope and accompanying components in sink 1 (with added detergent).
- More detailed cleaning instructions are available in department SOP’s.

Rinse Scope

- Rinse scope and accompanying components in clean, fresh water in sink 2.
- Rinsing necessary to remove detergent to avoid frothing in following machine cleaning process.

Freq:	Manually Leak Test Scope	Add Detergent to Sink	Clean Outside Surface of Scope	Rinse Scope
	Per scope	Per scope	Per scope	Per scope

SC3.0 Prepare Scope for Machine Cleaning

Technician

- This is the process of finalising the manual scope cleaning stages and preparing a manually cleaned scope for machine cleaning.
- This process map does not include all steps such as replenishment of machine consumables, the machine cleaning itself, or removal of scopes from the machine after the machine cleaning cycle.
- The process assumes an AER (machine) is open and available.
- The process ends assuming an AER has been filled to its 3 scope capacity.

Key Drivers:
Scopes
AER

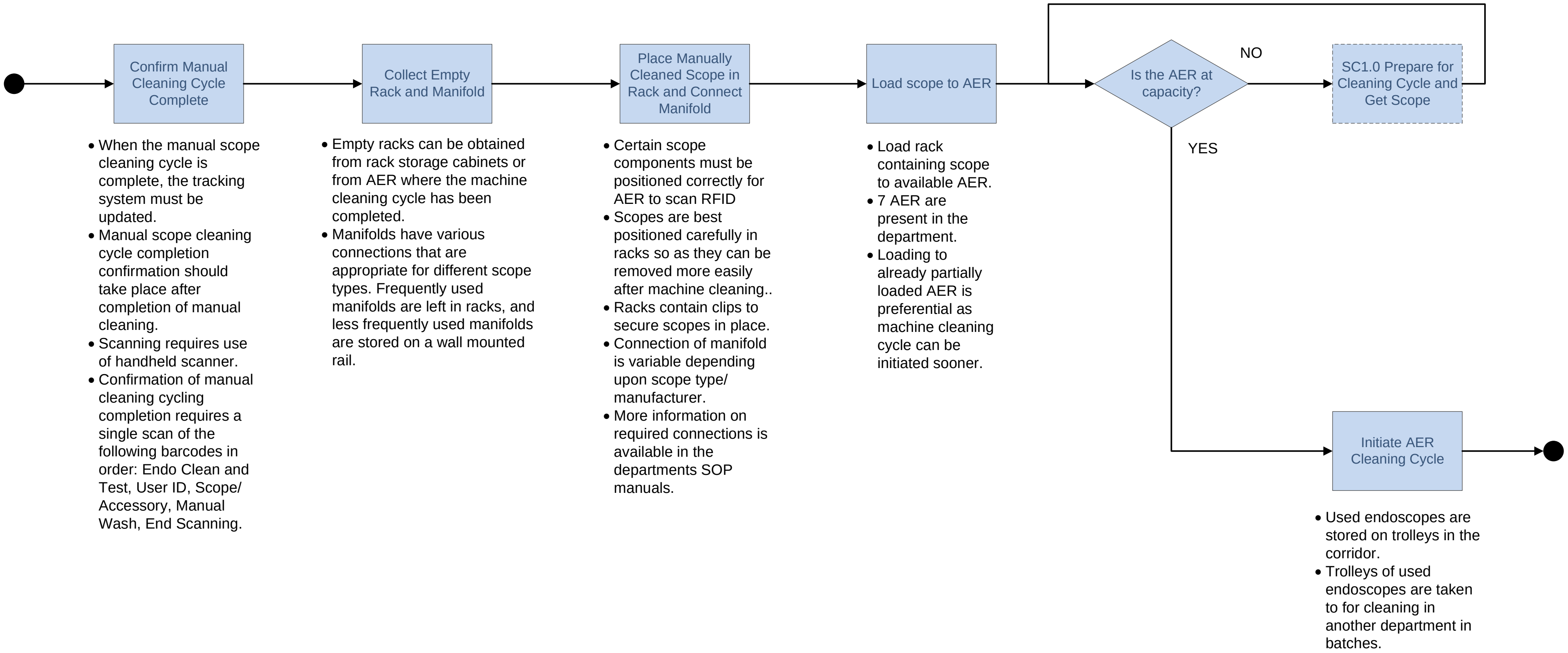

Freq:					
	Per scope	Per scope	Per scope	Per scope	Per AER
